# DNA Damage Estimation after Chronic and Combined Exposure to Endocrine Disruptors: An In Vivo Real-Life Risk Simulation Approach

**DOI:** 10.3390/ijms24129989

**Published:** 2023-06-10

**Authors:** Vasiliki Karzi, Eren Ozcagli, Manolis N. Tzatzarakis, Elena Vakonaki, Irene Fragkiadoulaki, Aikaterini Kalliantasi, Christina Chalkiadaki, Athanasios Alegakis, Polychronis Stivaktakis, Aikaterini Karzi, Antonios Makrigiannakis, Anca Oana Docea, Daniela Calina, Aristidis Tsatsakis

**Affiliations:** 1 Laboratory of Toxicology, Medicine School, University of Crete, 70013 Heraklion, Greece; chemstud.vas2010@gmail.com (V.K.); tzatzarakis@uoc.gr (M.N.T.); evakonaki@gmail.com (E.V.); eirinimbg@hotmail.gr (I.F.); kalliantash_k@hotmail.com (A.K.); christina4495@hotmail.com (C.C.); alegkaka@uoc.gr (A.A.); polychronis.stivaktakis@gmail.com (P.S.); katerinakarzi@hotmail.gr (A.K.); 2 Department of Pharmaceutical Toxicology, Faculty of Pharmacy, Istanbul University, Beyazıt, Istanbul 34116, Turkey; eren.ozcagli@istanbul.edu.tr; 3 Department of Obstetrics and Gynecology, University Hospital of Heraklion, 71500 Heraklion, Greece; makrygia@uoc.gr; 4 Department of Clinical Pharmacy, University of Medicine and Pharmacy of Craiova, 200349 Craiova, Romania; daoana00@gmail.com; 5 Department of Toxicology, University of Medicine and Pharmacy of Craiova, 200349 Craiova, Romania; calinadaniela@gmail.com

**Keywords:** glyphosate, bisphenol a, parabens, bis (2-ethylhexyl) phthalate, triclosan, rabbits, comet assay, micronuclei assay

## Abstract

Exposure to chemical substances has always been a matter of concern for the scientific community. During the last few years, researchers have been focusing on studying the effects resulting from combined exposure to different substances. In this study, we aimed to determine the DNA damage caused after chronic and combined exposure to substances characterized as endocrine disruptors using comet and micronuclei assays, specifically glyphosate (pure and commercial form), bisphenol A, parabens (methyl-, propyl- and butylparaben), triclosan and bis (2-ethylhexyl) phthalate. The highest mean tail intensity was observed in the group exposed to a high-dose (10 × ADI) mixture of substances (Group 3), with a mean value of 11.97 (11.26–13.90), while statistically significant differences were noticed between the groups exposed to low-dose (1 × ADI) (Group 2) and high-dose (10 × ADI) (Group 3) mixtures of substances (*p* = 0.003), and between Group 3 and both groups exposed to high doses (10 × ADI) of the pure and commercial forms of glyphosate (Groups 4 (*p* = 0.014) and 5 (*p* = 0.007)). The micronuclei assay results were moderately correlated with the exposure period. Group 5 was the most impacted exposure group at all sampling times, with mean MN counts ranging between 28.75 ± 1.71 and 60.75 ± 1.71, followed by Group 3 (18.25 ± 1.50–45.75 ± 1.71), showing that commercial forms of glyphosate additives as well as mixtures of endocrine disruptors can enhance MN formation. All exposure groups showed statistically significant differences in micronuclei counts with an increasing time trend.

## 1. Introduction

The continuous increase in the population and the over-consumerism that is currently being promoted have raised the use of agrochemicals and preservatives in order to increase food production and ensure food quality. However, this phenomenon has led to the constant exposure of the population to a variety of compounds that are marketed by manufacturers as harmless, with little or no side effects. Most of the existing studies aim to estimate the side effects of exposure to one chemical, rather than determining the synergic effects of the combined exposure that actually happens in real life. The same applies to the existing authorized guidelines and regulations, which are focused on one substance or one group of substances [1,2]. The increase in the amount of data from biomonitoring studies has caused researchers to turn their attention to the synergic or antagonistic effects of exposure to more than two compounds, introducing the concept of real-life risk simulation (RLRS) [1,2,3,4]. 

Glyphosate (GLY) is the active substance of one of the most used pesticides, Roundup. Although GLY has been proven to be toxic, it is noteworthy that its commercial form, Roundup, presents excessive toxicity due to its adjuvants, especially polyethoxylated tallow amine (POEA) [5,6]. In the last decade, there has been a large debate concerning the detrimental effects of exposure to this pesticide and the proven association with various forms of cancer, kidney and liver damage, delivery problems, mental conditions and DNA damage [7]. However, Roundup is still classified by regulatory agencies as safe due to the lack of significant studies showing its toxicity, and its residues are present in the environment, thus entering the food chain [8]. 

Parabens (PBs) and triclosan (TCS) are antimicrobial substances widely used in personal care products (PCPs). It has been shown that both PBs and TCS can disrupt the regular function of certain hormones, especially those related to reproduction such as estrogens, androgens and testosterone [9,10]. PBs are also related to reproductive problems, breast cancer, obesity, genotoxicity and allergies, while TCS is characterized as an allergen [9].

Bisphenol A (BPA) and bis (2-ethylhexyl) phthalate (DEHP) are plasticizers used in a variety of products, but the main source of exposure is food packaging and plastic bottles [11,12]. BPA is used for the production of epoxy resins coating the inner layer of packaging [13], while DEHP is used for the production of polyvinyl chloride (PVC), in order to provide softer and more flexible materials [11]. Both are proven to act like endocrine disruptors, thus affecting several systems of the organism, such as the reproductive system, immune system, metabolism and development [11,12]. 

In the last few years, exposure studies have been focusing on determining DNA damage since it strongly relates to or even causes all proven problems [14]. Exposure of the African catfish to glyphosate triggered the formation of micronuclei abnormalities and other nuclear abnormalities such as bean-shaped cells, while the comets formed in the analyses of the liver cells indicated potential genotoxic properties [15]. Previously, experiments by Koller et al. in a buccal epithelial cell line proved that exposure to glyphosate and/or Roundup led to elevated frequencies of micronuclei and nuclear buds and increased membrane damage [5]. 

Studies on EDC mixtures have indicated elevated effects of co-exposure in contrast with mono-exposure to EDCs [16,17]. Treatment of HepG2 cells with binary mixtures of BPA, dibutyl phthalate (DBP) and cadmium had a stronger DNA damage effect than individual exposure, while stronger cytotoxicity and the generation of greater amounts of reactive oxygen species (ROS) were also observed [18]. Likewise, enhanced ROS production and, hence, oxidative stress biomarker variations were noticed by Falfushynska and co-authors in zebrafish administered a mixture of chlorpyrifos and Roundup. Additionally, decreased cortisol levels were measured after combined exposure to a mixture of the aforementioned pesticides [19]. It is worth noting that Dinca et al. found cytotoxic and histopathological effects even at very low doses (below NOAEL—No Observed Adverse Effect Level) in rats exposed to a mixture of chemical substances containing several pesticides, ethylparaben, butylparaben, bisphenol A and others [20].

The objective of this study was to investigate the DNA damage caused by chronic and combined exposure to substances characterized as endocrine disruptors using two different techniques that offer different approaches to the issue. The comet assay detects the fragmentation of DNA, while the micronuclei assay detects chromosome breakage and impairment of the mitotic apparatus [21]. Both methods have been previously applied and proven to be reliable for the detection of DNA damage in human and animal cells caused by various chemical substances [22,23,24]. Specifically, we aimed to determine the extent of DNA damage in peripheral lymphocytes and tissues in rabbits after 12 months of exposure to GLY (pure and commercial forms), BPA, methylparaben (MePB), propylparaben (PrPB), butylparaben (BuPB), TCS and DEHP.

## 2. Results

### 2.1. Micronuclei Counts

**Figure 1:**
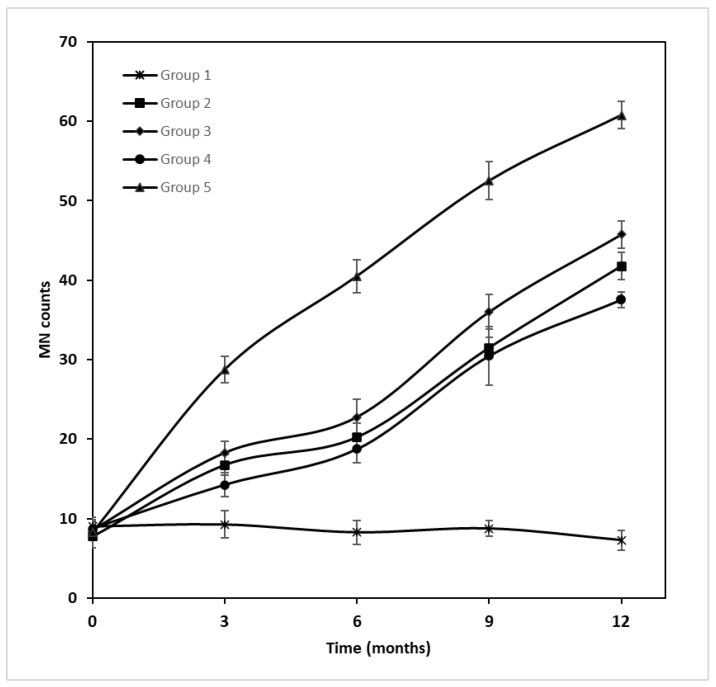
MN expression in different exposure groups at different sampling times.

Repeated measures ANOVA using the exposure groups as factors was applied for MN counts at 0, 3, 6, 9 and 12 months of exposure, to estimate both time and exposure effects. A statistically significant difference was observed in the time and exposure interaction (ANOVA F(1, 4) = 479.23, *p* < 0.001). The group details are presented in Table 1. The Group 1 values were stable at the experiment time points, ranging from 7.25 ± 1.26 (12 months) to 9.25 ± 1.71 (3 months). All other groups showed an increasing time trend, indicating time dependence on micronuclei formation. In Group 2, 7.75 ± 0.50 was measured at t = 0 months, which increased to a mean value of 41.75 ± 1.71 in the 12th month. Group 3 seemed to increase in a stable pattern from 8.50 ± 1.00 (t = 0 months) to 45.75 ± 1.71 (t = 12 months). Similarly, Group 4 and Group 5, starting from 8.75 ± 0.96 and 8.25 ± 1.89, reached values of 37.50 ± 1.00 and 60.75 ± 1.71, respectively (Table 2 and Figure 1). Post hoc comparisons (LSD test) showed a statistically significant difference at the *p* < 0.001 level when the total group mean values were compared. 

Group 3 was administered a 10 times higher dose than Group 2; however, the MN counts did not present proportional values. Over the sampling times t = 3–12 months, Group 2 MN counts ranged from 16.75 ± 1.26 to 41.75 ± 1.71, while those of Group 3 slightly differed, with mean MN counts of 18.25 ± 1.50–45.75 ± 1.71. Group 4 was administered the same dose of GLY as Group 3, but Group 3 also contained a mixture of endocrine disruptors. Group 3 appeared to be more burdensome than Group 4 (18.25 ± 1.50–45.75 ± 1.71 and 14.25 ± 1.50–37.50 ± 1.00, respectively), which could be attributed to the additional endocrine disruptors administered to Group 3. Although Groups 4 and 5 did not differ in dose, the adjuvants in the commercial form of GLY seemed to affect further MN formation, while Group 5 had higher mean values of MN (28.75 ± 1.71–60.75 ± 1.71 to 14.25 ± 1.50–37.50 ± 1.00 of Group 4), in the same periods of exposure. Additionally, Group 5 had the highest mean MN counts of all groups.

### 2.2. Tail Intensity

**Figure 2:**
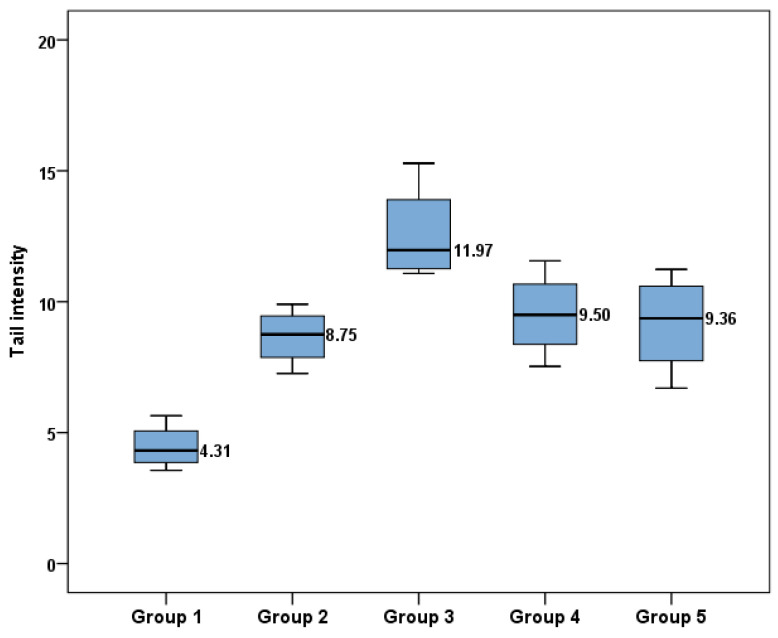
Mean tail intensity values between groups at 12 months of exposure.

The tail intensity was measured using blood from the last sampling (t = 12 months). All exposure groups seemed to be affected in relation to Group 1, which was the control group. As shown in Figure 2, the tail intensity of each group was significantly different (ANOVA: F(4, 15)= 13,974, *p* < 0.001, Kruskal–Wallis χ2(4) = 13.786, *p* = 0.008). The highest median tail intensity, 11.97 (11.26–13.90), was observed in Group 3, while the intensity in Group 1 was 4.31 (3.86–5.06) (Table 3), showing that combined exposure to endocrine disruptors had a greater impact on DNA damage and, by extension, the tail intensity. 

The difference in the tail intensity mean values between Group 2 (8.75; 7.88–9.46) and Group 3 (11.97; 11.26–13.90) is proof of a dose-dependent response. Interestingly, the mean tail intensity values of Groups 4 and 5 were similar (9.50; 8.37–1.67 and 9.36; 7.74–10.59, respectively), which is evidence that the adjuvants of the commercial form of GLY do not enhance breaks in the DNA. Statistically significant differences were obtained from the LSD adjusted t-test. Between Groups 2 and 3, which had different levels of exposure, a significant difference was observed (*p* = 0.003), while Group 3 was significantly different from Group 4 (*p* = 0.014) and Group 5 (*p* = 0.007) at t = 12 months (Table 3).

### 2.3. Correlation of Micronuclei and Comet Assay Results

**Figure 3:**
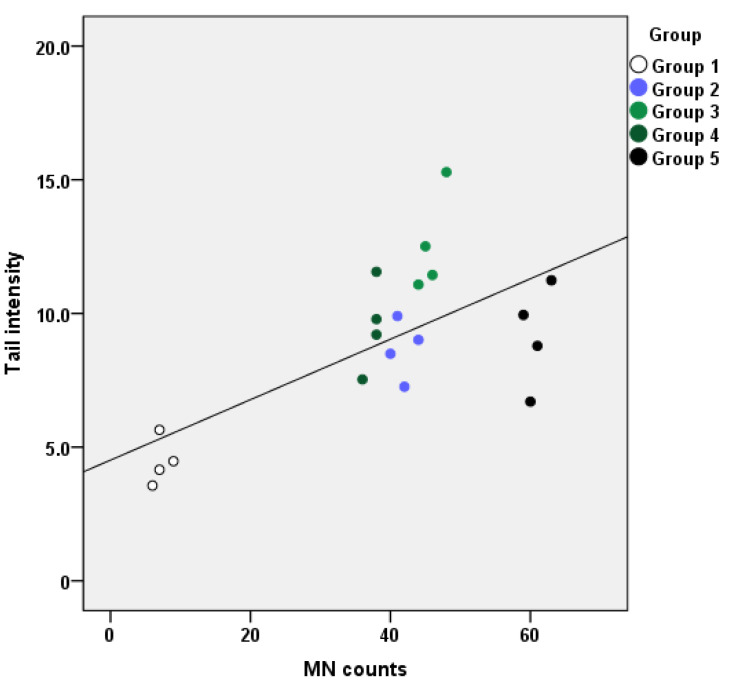
Correlation of tail intensity with micronuclei counts (MN) for each of the five different groups at t = 12 months (rs = 0.581, *p* = 0.007).

MN counts at t = 12 months were correlated with the corresponding tail intensity results. All exposure groups’ MN counts appeared to be statistically significantly associated with the tail intensity, proving the genotoxicity of the administered substances. In Figure 3, scatterplots of the tail intensity vs. MN counts at 12 months are presented. The baseline MN counts were correlated with the tail intensity (rs = 0.581, *p* = 0.007).

In Table 4, measurements of the MN counts at different time points of exposure are shown. The baseline counts were not significantly correlated between the time intervals (*p* > 0.05). All other bivariate correlations of the MN counts were strongly correlated, with the lowest r value observed between the 3- and 6-month exposure counts (rs = 0.826. *p* < 0.001).

## 3. Discussion

In the current study, we aimed to determine DNA damage caused by chronic exposure to substances characterized as endocrine disruptors. Specifically, we tried to simulate the real-life exposure by administering GLY, TCS, BPA, MePB, PrPB, BuPB and DEHP to rabbits at different doses for 12 consecutive months. 

The micronuclei assay showed significant differences from the beginning to the end of the exposure period. Except for Group 1, which showed similar mean values throughout the whole experiment (7.25 ± 1.26–9.25 ± 1.71), all other groups showed differences in a time- and dose-dependent manner. At the last time point of exposure (12 months), the mean MN values of Groups 2 and 3 (41.75 ± 1.71 and 45.75 ± 1.71, respectively) seemed to be five times the initial values (7.75 ± 0.50 and 8.50 ± 1.00, respectively), while the mean MN value of Group 4 seemed to three times the initial value at t = 0 months (from 8.75 ± 0.96 to 37.50 ± 1.00). The most affected group seemed to be Group 5, which showed a 7 times higher value at 12 months (60.75 ± 1.71) in comparison with that at t = 0 months (8.25 ± 1.89).

Koller et al. studied the cytotoxic and genotoxic properties of GLY and Roundup in a buccal epithelial cell line, observing that Roundup was more active than GLY and induced cytotoxic effects due to membrane damage and impairment of mitochondrial functions [5]. This observation can be found in our results, as Group 5’s (commercial GLY exposure group) MN counts at the end of the in vivo experiment (t = 12 months) were almost twice as much as those of Group 4 (pure GLY exposure group). The commercial form of GLY (Roundup) contains additives including the adjuvant POEA, which is proven to cause excessive genomic damage [6], a fact that may justify the difference between Groups 4 and 5. 

At all sampling time points, Group 3 (8.50 ± 1.00–45.75 ± 1.71) presented higher MN counts than Group 2 (7.75 ± 0.50–41.75 ± 1.71), but this did not correspond to the tenfold difference in the administered dosage. Interestingly, Group 3 presented greater MN counts than Group 4 (8.75 ± 0.96–37.50 ± 1.00), although they were not statistically significant. This difference in MN measurements is attributed to the extra substances (BPA, TCS, DEHP, MePB, PrPB and BuPB) that Group 3 contained in relation to Group 4 (pure GLY), concluding that there is a synergic additive effect of GLY with these compounds in MN counts. 

The results from previous studies vary depending on the substances of exposure. Duarte et al. observed increased MN frequencies when hepatic cells were exposed to TCS and DEHP separately. However, when exposed in combination, no induced micronuclei formation was observed, while cell viability seemed to be negatively affected [25]. On the other hand, experiments on the exposure of hepatocytes to a mixture of bisphenols revealed increased cytotoxicity with MN formation, but no such effects were observed when hepatocytes were exposed to individual bisphenols [26]. 

It is worth noting that Group 5 was the most impacted exposure group concerning MN formation (8.25 ± 1.89–60.75 ± 1.71), where it had almost twice the MN counts of Group 4 (8.75 ± 0.96–37.50 ± 1.00). Although Groups 4 and 5 were administered the same dose of GLY, the former contained pure GLY, while the latter contained all the adjuvants included in the commercial form of GLY (Roundup). One of these adjuvants is POEA, the toxicity of which has been proven in animal experiments [8,27]. Notably, the results retrieved from experiments on juvenile fish indicated its ability to be genotoxic, induce lipid peroxidation and unbalance the redox status [28]. 

The comet assay results showed that Group 3 had a higher tail intensity value (12.58 ± 1.90), which was significantly different from that of Group 2 (8.67 ± 1.10), suggesting a dose-dependent damage, as these two groups had a tenfold difference in the administered dosage. The same trend was observed in previous studies when fish and human lymphocytes were exposed to GLY, TCS and PBs individually [15,29,30]. Groups 4 and 5 showed no significant difference, indicating that the additives of the commercial form of GLY had no additive effect on the tail intensity. However, Groups 3 and 4 presented statistically significant differences in the mean tail intensity values (12.58 ± 1.90 and 9.52 ± 1.66, respectively), indicating a synergic effect of GLY, BPA, TCS, DEHP, MePB, PrPB and BuPB on the tail intensity. Previously, comet assay tests on murine cell lines exposed to GLY, Roundup and POEA showed that the inhibitory potency followed the order POEA > Roundup > GLY [31]. The differences in bibliographic data can be explained by the limited number of studies and the diversified exposure groups. 

The correlation of the tail intensity with the MN counts at 12 months of exposure showed statistically significant associations among the exposure groups (*p* < 0.001). Both the tail intensity and MN counts showed a time-dependent response, while some of the groups also responded in a dose-dependent manner. In previous studies, the target compounds GLY, BPA, TCS, DEHP and PBs seemed to have detrimental effects on the MN counts, which increased with increasing exposure period and dose [3,21,30,32,33], but the tail intensity did not respond to this trend in the same capacity [6]. 

All of the target substances of the current study seemed to present genotoxic activity. In previous studies, TCS has shown a dose-dependent genotoxic effect in different organisms, gradually increasing the tail intensity and micronuclei formation [29,34] or even leading to complete nuclei dissolution at high exposure doses [35]. In vitro experiments have proven that PBs’ cytotoxic and genotoxic effects induce MN formation and increase tail intensity in a concentration-dependent manner [30]. Both phthalates and BPA have also been proven to be genotoxic and negatively affect chromosomes and tail length, in a dose-dependent manner [36,37,38,39]. Furthermore, the induced DNA damage and cellular proliferation have been attributed to DEHP exposure. In particular, exposure of rats to DEHP led to elevated comet tail moments in both the cells and thyroid tissue of juvenile rats [40].

Similar research studying the genotoxic effects of pesticides observed significant differences with increasing exposure time [41,42]. Vardavas et al. noticed significant increases in binucleated MN after the exposure of rabbits to cypermethrin. The induced genotoxicity was accompanied by histopathological lesions as well as liver and kidney inflammation [41]. Likewise, Stivaktakis et al. found differences in the MN counts between control groups and groups of rabbits exposed to imidacloprid, but there was no time dependence of the genotoxic effect [42]. This fact can be explained by the application of detoxification mechanisms, possible metabolization of the xenobiotics and DNA damage repair [43].

For the sake of argument, micronuclei formation and generation of DNA strand breaks directly affect the functions of cells, provoking problems in the organism [14]. Our results indicate that mixtures of EDCs have a greater negative impact on DNA, a fact that emphasizes the danger posed by daily exposure to these substances and the need for further research in this field.

## 4. Materials and Methods

### 4.1. In Vivo Experiment

The in vivo experiment has been described in detail previously [44]. In brief, 20 New Zealand albino rabbits, 10 males and 10 females, 3–4 months old, weighing approximately 3 kg each, were divided into 5 treatment groups of 4 animals each. The details of the groups and doses are presented in Table 1. The substances were orally administered to the rabbits once a day, 7 days per week, for 12 months. Solutions of the substances dissolved in 5% ethanol/water were prepared and mixed with the rabbits’ food in specific amounts (final daily amount of ethanol was less than 0.5%). The dose calculation was based on the established acceptable daily intakes (ADIs) of the substances set by authorized services. The control group (Group 1) received a normal diet (water, pellets, corn oil with 5% ethanol/water). This research has been registered in the Animal Study Registry (DOI: 10.17590/asr.0000259). The protocol was approved by the Committee of Ethics and Academic and Scientific Deontology of the University of Medicine and Pharmacy of Craiova (4/17.01.2020).

### 4.2. Reagents

GLY (PESTANAL^®^, analytical standard, CAS number: 1071-83-6), MePB (Methyl 4-hydroxybenzoate) (ReagentPlus^®^, ≥99.0%, crystalline, CAS number: 99-76-3), PrPB (Propyl 4-hydroxybenzoate) (≥99%, CAS number: 94-13-3), BuPB (Butyl 4-hydroxybenzoate) (≥99.0% (GC), CAS number: 94-26-8), BPA (>99%, CAS number: 80-05-7) and DEHP (PESTANAL^®^, analytical standard, CAS Number: 117-81-7) were purchased from Sigma-Aldrich (St. Louis, MO, USA). TCS (100%) and ethanol absolute (≥99.8%, CAS number: 64-17-5) were obtained from Honeywell–Fluka (Seelze, Germany), methanol (≥99.8%, CAS number: 67-56-1) was obtained from Honeywell–Riedel de Haën (Seelze, Germany) and acetic acid glacial (≥99.7%, CAS Number: 64-19-7) was obtained from VWR (Radnor, PA, USA). 

DPX mountant for histology was purchased from Sigma-Aldrich (MO, USA), while KaryoMAX™ Giemsa Stain Solution (CAS Number: 67-56-1) and PHA (Ref. number: 10576-015) were purchased from Gibco™ (Dreieich, Germany). FBS and HAM were obtained from Biowest (Nuaillé, France), and cytochalasin b (CAS number: 14930-96-2) was obtained from Acros Organics (Geel, Belgium). Ultrapure water (ddH2O) was produced using a Direct-Q 3UV water purification system (Merck, Darmstadt, Germany). 

Dimethyl sulfoxide (for molecular biology, Cas number: 67-68-5), ethidium bromide (95% HPLC, CAS No.: 1239-45-8), sodium chloride (≥99% (titration), CAS No.: 7647-14-5), Na_2_EDTA (98.5–101.5% (titration), CAS No.: 6381-92-6), trizma base (≥99.9% (titration), CAS Number: 77-86-1), phosphate-buffered saline (tablet), TritonTM X-100 (laboratory grade, CAS Number: 9036-19-5) and trypan blue test (0.4%, for microscopy, CAS Number: 72-57-1) were purchased from Sigma-Aldrich (Gillingham, UK).

Finally, NMA (Catalog number: 16500100) and LMA (Catalog number: 16520100) were obtained from Thermo Fisher Scientific (Waltham, MA, USA).

The acceptable daily intake (ADI) for various chemicals is determined by the European Food Safety Authority (EFSA) and the related service in Canada. The EFSA has set the ADI to 0.004 mg/kg of bw/day for BPA [45], 0.5 mg/kg of bw/day for BuPB, 5 mg/kg of bw/day for MePB and 5 mg/kg of bw/day for PrPB [46]. The ADI for total phthalates is up to 0.05 mg/kg of bw/day [47], and the ADI for glyphosate is up to 0.5 mg/kg of bw/day [48]. The Environment and Climate Change Canada and the Health Canada services have defined the ADI for TCS as 0.08 mg/kg of bw/day [49].

### 4.3. Micronuclei Assay

The micronuclei assay procedure has been described in detail previously [22,50]. The sampling was conducted every 3 months (t = 0, 3, 6, 9, 12 months). Blood samples (from the jugular vein) were collected in heparin tubes and stored at 4 °C until analysis. In brief, 0.5 mL of each blood sample was mixed with 6.5 mL of HAM medium, 1.5 mL of FBS, 0.3 mL of phytohemagglutinin (PHA) and 0.1 mL of antibiotic. The cell culture was prepared under sterile conditions in special cell culture flasks and placed into an incubator at 37 °C with a 5% CO_2_ supply. After incubation for 44 h, 20 μL of cytochalasin b was added to each cell culture, which was then placed back into the incubator for 28 h (72 h of incubation in total). 

The next step was cell fixation, firstly with 4 mL of hypotonic solution (ddH_2_O:HAM 1:1) and then with 4 mL of fixative solution (CH_3_OH:CH_3_COOH 3:1). Washes with fixative solution were repeated until the liquid was clear. After the final centrifugation, most of the supernatant was removed, and the pellet was gently resuspended with the 0.5 mL of supernatant that remained. The cells were then placed on slides by dripping two drops of the solution on each slide. For each cell culture, 3 slides were prepared. The slides were left to air-dry overnight.

The slides were then submerged in 15% Giemsa solution for 20 min, followed by ddH_2_O for a few seconds immediately after, and finally left to air-dry for 2 h. When the slides were completely dry, DPX mountant was used to glue the coverslips to each slide. The slides were left to air-dry overnight and were then ready for microscope measurement.

Slides were examined with an optical microscope at 100× magnification using cedar oil as the immersion oil. For each animal, micronuclei were scored in 1000 binucleated cells. The number of binucleated cells that contained micronuclei was also counted. The scoring criteria for selecting binucleated cells that can be scored for MN frequency were described in detail by Michael Fenech [51]. In order to determine additional possible cytotoxic effects, the cytokinesis-block proliferation index (CBPI) was also calculated. Counting 2000 cells and based on the equation CBPI = [M_1_ + 2M_2_ + 3 (M_3_ + M_4_)]/N, where M_1_, M_2_, M_3_ and M_4_ correspond to the number of cells with one, two, three and four nuclei, respectively, and N is the total number of cells, the CBPI is a useful index for determining additional cytotoxic effects [52].

### 4.4. Comet Assay

Venous blood samples (from the jugular vein) were collected during the last sampling (t = 12 months) in tubes containing ethylenediaminetetraacetic acid (EDTA), and then 200 μL aliquots were stored at −80 °C. After the addition of 1 mL cold phosphate-buffered saline (PBS) to 100 μL whole blood, lymphocyte isolation was achieved using lymphocyte separation medium at the bottom of the tube and centrifuged immediately at 4 °C for 3 min at 200× *g*. Lymphocytes were obtained from the layer that appeared between the blood plasma and the lymphocyte separation medium after centrifugation. The cell viability, as assessed via the trypan blue test, was more than 97% for all samples. Each analysis was conducted in triplicate for this study.

The alkaline comet assay technique has been previously described in detail [53,54,55]. Shortly after the preparation of the slides, they were treated with 0.65% normal melting agarose (NMA) prepared in PBS (Ca^2+^- and Mg^2+^-free). Isolated lymphocytes were mixed with 100 μL LMA at 37 °C to form a cell suspension. After gently removing the coverslip, the slides were immersed in cold lysing solution (2.5 M NaCl, 100 mM Na_2_EDTA, 10 mM Tris, pH = 10) including 1% Triton X and 10% DMSO overnight. 

The slides were then removed from the lysing solution, drained and placed in a horizontal gel electrophoresis tank near the anode. Electrophoresis was performed at 1.6 V/cm for 20 min (300 mA) at 4 °C room temperature under dimmed light.

After electrophoresis, the slides were taken from the tank. Tris buffer (0.4 M Tris, pH 7.5) was gently added drop-wise, and the slides were allowed to sit for 5 min; this procedure was repeated three times. 

Each slide was stained with 50 μL ethidium bromide and stored in a humidified air-tight container until being analyzed within 3–4 h. In total, 3 slides prepared for each subject and 100 cells per subject were analyzed at ×400 magnification, under an Olympus fluorescent microscope equipped with an excitation filter of 546 nm and a barrier filter of 590 nm. The Comet Assay IV image analysis system (Perceptive Instruments) was used blindly by one slide reader in order to score DNA damage. The analysis software (Comet Assay IV, https://www.instem.com/solutions/genetic-toxicology/comet-assay.php, accessed on 12 May 2023) provides automated analysis of comet size, shape and the amount of DNA that has been formed due to the fragmentation. Specifically, it provides a range of parameters including the tail length, tail intensity and tail moment [56]. The tail intensity is defined as the percentage of DNA that migrates to the tail, and it is a widely favored measurement for the comet assay because of its advantages. Therefore, this parameter was selected for measuring DNA damage at the single-cell level [56]. 

### 4.5. Statistical Methods

The tail intensity is expressed as the mean and standard deviation, while median values are also used. One-way analysis of variance (one-way ANOVA) was applied to examine differences in the intensity or micronuclei counts between exposure groups, while additional Kruskal–Wallis analyses were performed. Spearman’s r_s_ coefficients were applied to examine associations between the intensity and micronuclei counts. LSD (least square differences) adjusted t-tests followed by ANOVA were performed for the pairwise comparisons between groups. Repeated measures ANOVA using the exposure groups as factors was applied to establish associations between micronuclei counts at baseline and 3, 6 and 12 months after the experiment and the glyphosate exposure groups. 

IBM SPSS Statistics 24.0 was used for statistical analysis of the data, and a = 0.05 was set as the level of significance.

## 5. Conclusions

In the last five years, the European Union has made substantial efforts in EDC testing and screening methods to identify endocrine-disrupting chemicals, coordinating a cluster group of eight research projects (EURION) [57]. In the current study, we aimed to determine the DNA damage caused to rabbits’ lymphocytes after 12-month exposure to GLY, BPA, MePB, PrPB, MePB, TCS and DEHP using two different approaches: micronuclei assay and comet assay. The results from the different samplings through the in vivo experiment showed that MN formation increased in a dose- and time-dependent manner. The same also applied to the tail intensity, but this was only related to the dose, as there was only one sampling (the last one). Group 3 seemed to be the most affected regarding the tail intensity, probably due to the synergic effect of the administered substances. In all exposure groups, the MN counts were positively associated with the tail intensity. This study is one of the few that have attempted to identify the risks that exposure to both low and high dose levels of EDC mixtures pose to humanity. There is a great way forward for science in order to thoroughly investigate the real-life risks.

## Figures and Tables

**Table 1:** Groups and doses of the in vivo experiment.

Group		Dose	Substance
1	Control	-	-
2	Low-dose mix	1 × ADI	GLY, BPA, MePB, PrPB, BuPB, TCS, DEHP
3	High-dose mix	10 × ADI	GLY, BPA, MePB, PrPB, BuPB, TCS, DEHP
4	High-dose pure GLY	10 × ADI	GLY
5	High-dose commercial GLY	10 × ADI	GLY

ADI is 0.004 mg/kg bw/day for BPA; 0.5 mg/kg bw/day for BuPB; 0–10 mg/kg bw/day for MePB and PrPB; up to 0.05 mg/kg bw/day for total phthalates; and up to 0.5 mg/kg bw/day for glyphosate (EFSA values). ADI for TCS is 0.08 mg/kg bw/day (Government of Canada values).

**Table 2:** Mean and standard deviation of MN counts at different time points in different exposure groups.

Months	Group 1		Group 2		Group 3		Group 4		Group 5	
	Mean	±SD	Mean	±SD	Mean	±SD	Mean	±SD	Mean	±SD
0	9.00	0.82	7.75	0.50	8.50	1.00	8.75	0.96	8.25	1.89
3	9.25	1.71	16.75	1.26	18.25	1.50	14.25	1.50	28.75	1.71
6	8.25	1.50	20.25	1.71	22.75	2.22	18.75	1.71	40.50	2.08
9	8.75	0.96	31.50	1.29	36.00	2.16	30.50	3.70	52.50	2.38
12	7.25	1.26	41.75	1.71	45.75	1.71	37.50	1.00	60.75	1.71
**Total mean**	8.50	1.25	23.60	1.29	26.25	1.72	21.95	1.77	38.15	1.95

Time × group interaction: F (1, 4) = 479.23, *p* < 0.001.

**Table 3:** Descriptive statistics (mean, SD, quartiles) and comparison of tail intensities between groups.

Group	Mean	±SD	Quartile			LSD Test				
			1st	Median	3rd	Group 1	Group 2	Group 3	Group 4	Group 5
1	4.46	0.88	3.86	4.31	5.06		0.002	<0.001	<0.001	0.001
2	8.67	1.10	7.88	8.75	9.46	0.002		0.003	0.449	0.655
3	12.58	1.90	11.26	11.97	13.90	<0.001	0.003		0.014	0.007
4	9.52	1.66	8.37	9.50	10.67	<0.001	0.449	0.014		0.752
5	9.17	1.92	7.74	9.36	10.59	0.001	0.655	0.007	0.752	

ANOVA: F (4, 15) = 13.974, *p* < 0.001.

**Table 4:** Correlation of micronuclei counts between months of exposure.

		Spearman’s r	
Months	Months	rs	*p*
0	3	−0.350	0.130
	6	−0.201	0.394
	9	−0.285	0.223
	12	−0.368	0.110
3	6	0.874	<0.001
	9	0.826	<0.001
	12	0.940	<0.001
6	9	0.928	<0.001
	12	0.887	<0.001
9	12	0.889	<0.001

## Data Availability

The data presented in this study are available on request from the corresponding author. The data are not publicly available due to ethical and privacy restrictions.
